# Increased expression of miR-601 is associated with poor prognosis and tumor progression of gastric cancer

**DOI:** 10.1186/s13000-019-0882-5

**Published:** 2019-09-23

**Authors:** Cuili Min, Aixia Zhang, Jing Qin

**Affiliations:** Department of Laboratory Medicine, Yidu Central Hospital of Weifang, No.4138, South Linglongshan Road, Shandong, 262500 China

**Keywords:** MicroRNA-601, Prognosis, Progression, Gastric cancer

## Abstract

**Background:**

MicroRNAs (miRNAs) have been considered to participate in many tumorigenesis, including gastric cancer (GC). Abnormal expression of miR-601 has been reported in GC, but its role is not clear. The goal of this study is to explore the expression patterns, clinical value and functional role of miR-601 in GC.

**Methods:**

Quantitative real-time polymerase chain reaction (qRT-PCR) was conducted to evaluate the expression level of miR-601. The association between miR-601 expression and overall survival was estimated by the Kaplan-Meier survival method. The significance of different variables with respect to survival was analyzed by using the Cox regression assay. Cell experiments were applied to investigate the functional role of miR-601 in GC.

**Results:**

We found that miR-601 was significantly up-regulated in GC tissues and cells compared with the controls (all *P* < 0.01). The levels of miR-601 expression were significantly associated with TNM stage, lymph node metastasis, lymphatic invasion, and distant metastasis (all *P* < 0.05). Kaplan-Meier survival analysis showed that patients in the high miR-601 expression group had poor overall survival (log-rank *P* = 0.001). Moreover, we confirmed that miR-601, TNM stage, and distant metastasis were independent prognostic factors for GC patients. Overexpression of miR-601 in AGS and SGC-7901 cells by miR-601 mimic transfection significantly promoted the cell proliferation, migration, and invasion (*P* < 0.05).

**Conclusions:**

The expression level of miR-601 is dramatically up-regulated in GC. The overexpression of miR-601 promotes the tumor progression of GC, and may be a novel prognostic factor for poor survival in GC patients.

## Introduction

Gastric cancer (GC) is one of the most common malignant tumors, and it has become the leading cause of cancer-related death [1, 2]. Although the incidence of GC has a dropping tendency worldwide, it remains higher in China [3, 4]. At present, surgery is the most common method of GC treatment, as well as a radical type of treatment [5]. In recent years, with the wide use of gastric endoscopy, the early diagnosis and treatment of GC have been improved [6]. However, as a result of the tumor recurrence, invasion, and metastasis, the prognosis of GC is still very poor. Thus, it is urgent to find novel potential molecule biomarkers for early diagnosis and accurate prediction of prognosis.

MicroRNAs (miRNAs), are a class of endogenous, conserved and small non-coding RNA molecules, and regulate gene expression at the post-transcriptional level via binding to the 3′- untranslated region (UTR) of target messenger RNAs (mRNAs) [7]. MiRNAs have been reported to be involved in multiple cellular processes, including cell cycle, apoptosis, metabolism, and metastasis [8]. Importantly, abnormal expression of numerous miRNAs has been detected in various cancers, including GC [9–11]. Qiu et al. showed that miR-671-5p was downregulated in GC and overexpression of miR-671-5p inhibited GC cell proliferation and promoted cell apoptosis, it may serve as a tumor suppressor via targeting URGCP [12]. The low expression of miR-376a was also identified in GC tissues, which showed a significant association with the poor survival of GC patients [13]. Recent research reported that miR-601 was identified to be upregulated in GC through miRNA microarray profiling [14], suggesting the potential role of miR-601 in tumorigenesis and development of GC. But its concrete clinical and functional role in GC progression have not been studied.

In this study, we examined miR-601 expression in a larger cohort of GC. And the association of miR-601 expression with the clinicopathologic features of GC patients and its prognostic value were explored.

## Materials and methods

### Patients and sample collection

We collected 139 paired fresh GC and matched adjacent normal tissues from 139 patients who underwent gastrectomy at Yidu Central Hospital of Weifang from June 2007 to July 2011. All samples were quickly frozen in liquid nitrogen immediately after surgical removal and stored at − 80 °C. All participants were diagnosed with GC by pathological examination after surgery according to the World Health Organization criteria. All cases did not receive radiotherapy or chemotherapy before their surgery. The clinicopathological features of 139 patients were recorded in Table 1. After surgery, all patients were followed-up by means of a 5- year follow-up survey, and the survival information was obtained by telephone. This study was reviewed and approved by the Ethics Committee of Yidu Central Hospital of Weifang. Written informed consent was obtained from each participant.

**Table 1:** Association of miR-601 with the clinicopathological features of gastric cancer patients

Features	Total No. *N* = 139	miR-601 expression		*P* values
		Low (n = 59)	High (n = 80)	
Age (Years)				
≤ 60	55	23	32	0.904
> 60	84	36	48	
Gender				
Male	77	31	46	0.561
Female	62	28	34	
Perineural invasion				
Absence	90	38	52	0.942
Presence	49	21	28	
Histological type				
Differentiated	84	35	49	0.818
Undifferentiated	55	24	31	
Tumor depth				
T1-T2	95	38	57	0.391
T3-T4	44	21	23	
TNM stage				
I-II	79	43	36	0.001**
III-IV	60	16	44	
Lymph node metastasis				
No	71	37	34	0.018*
Yes	68	22	46	
Lymphatic invasion				
Absence	59	31	28	0.039*
Presence	80	28	52	
Venous invasion				
Absence	63	32	31	0.070
Presence	76	27	49	
Distant metastasis				
Absence	116	54	62	0.028*
peritoneal metastasis	13	3	10	
hepatic metastasis	7	1	6	
other distant metastasis	3	1	2	

*P < 0.05; **P < 0.01

### Cell culture and transfection

Normal human gastric mucosa cells GES-1 and GC cell lines including AGS, HGC-27, SGC-7901, and MGC-803 were purchased from the Cell Bank of the Chinese Academy of Sciences (Shanghai, China). The four GC cell lines were cultured in RPMI 1640 medium (Invitrogen, Carlsbad, CA, USA) supplemented with 10% fetal bovine serum (FBS) in a humidified incubator with 5% CO_2_ at 37 °C.

The miR-601 mimic, miR-601 inhibitor and the corresponding negative controls of mimic and inhibitor (mimic NC and inhibitor NC) were synthesized and purified by Gene-Pharma (Shanghai, China). The GC cells were transferred with the miR-601 mimic, miR-601 inhibitor or the negative controls by using Lipofectamine 3000 reagent (Invitrogen, USA) following the manufacturer’s protocols. Total RNA was collected 48 h after transfection.

### RNA extraction and quantitative real-time polymerase chain reaction (qRT-PCR)

Total RNA in the tissues and cells were isolated using Trizol Reagent (Invitrogen, Carlsbad, CA, USA) based on the manufacturer’s protocol. Reverse transcriptions were performed by using miScript Reverse Transcription Kit (QIAGEN, Germany). qRT-PCR was carried out to estimate the expression of miR-601, using SYBR green I Master Mix kit (Invitrogen, Carlsbad, CA, USA) and 300 Real-Time PCR System (Applied Biosystems, USA). The relative expressions of all genes were normalized to that of internal control U6 using the comparative delta CT (2^−ΔΔCt^) method.

### MTT assay

An MTT assay was conducted for the analysis of GC cell proliferation. The stably transfected cells were seeded in a 96-well plate with the density of 5 × 10^4^ cells/well and cultured at 37 °C with 5% CO_2_ for 3 days. Subsequently, 20 μl of MTT (5 mg/ml; Sigma-Aldrich; Merck, Darmstadt, Germany) was added in each well every 24 h and then incubated for further 4 h, followed by addition of 150 μl of dimethyl sulfoxide (DMSO) (Sigma-Aldrich; Merck). The cell proliferation was evaluated by measuring the absorbance at 490 nm using a microplate reader. The MTT assay was repeated 3 times.

### Transwell assay

For the investigation of cell migration and invasion, a Transwell assay was performed using Transwell chambers (8 μm pore size, Corning, USA). The membranes using for invasion analysis were pre-coated with Matrigel (Corning, USA), while no coating of Matrigel to the membranes for the cell migration assay. The stably transfected cells (5 × 10^4^ cells/well) were seeded into the upper chamber with serum-free medium, while the lower chamber was full of 300 μl DMEM containing 10% FBS. Following incubation at 37 °C for 24 h, the number of cells in the lower chambers were counted using a light microscopy.

### Statistical analysis

Student’s *t* test or one-way ANOVA analysis was used for the difference analysis between groups. Association of miR-601 expression with clinicopathological features was estimated by chi-square test. Survival analysis was performed for the GC patients using Kaplan-Meier methods and log-rank test. To confirm the prognostic value of miR-601 in cancer patients, cox regression analysis was performed. All the data analyses were conducted using SPSS version 18.0 software (SPSS Inc., Chicago, IL) and GraphPad Prism 5.0 software (GraphPad Software, Inc., USA). The data were expressed as mean ± standard deviation (SD). *P* < 0.05 was considered to indicate a statistically significant difference.

## Results

### Overexpression of miR-601 in GC tissues and cell lines

**Fig. 1:**
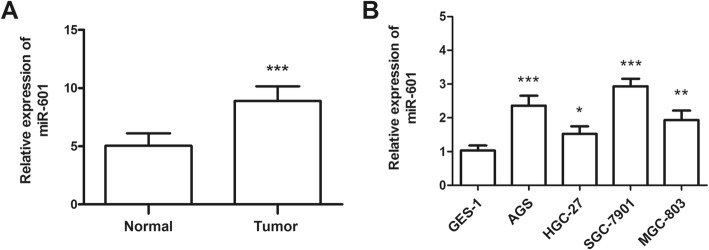
Expression of miR-601 measured by qRT-PCR in GC tissues and cell lines. **a** MiR-601 expression was upregulated in the GC tissues compared with the adjacent normal controls, which were calculated according to the mean value from 139 GC patients (*** *P* < 0.001). **b** The expression of miR-601 was higher in the GC cell lines than that in the normal gastric cells (* *P* < 0.05, ** *P* < 0.01 and ***P < 0.001)

The levels of miR-601 were estimated in GC tissues and four different GC cell lines. All the 139 paired GC tissues and adjacent normal tissues were subjected to qRT-PCR analysis. Results of the study indicated that the levels of miR-601 were significantly upregulated in GC tissues compared to adjacent normal tissues (*P* < 0.001, Fig. 1a). On analyzing miR-601 levels in the selected four GC cell lines (AGS, HGC-27, SGC-7901, and MGC-803), we found that miR-601 was upregulated in all the four cancer cell lines compared to normal GES-1cells, and the differences all reached the significant level (all *P* < 0.05, Fig. 1b).

### Association of miR-601 expression with clinicopathological features of GC patients

To explore the role of miR-601 clinically, we investigated the correlation between miR-601 and the clinicopathological characteristics of the enrolled GC patients. As shown in Table 1, the mean value of miR-601 was selected as the demarcation point, and the GC patients were classified into low expression group (*n* = 59) and high expression group (*n* = 80). The results revealed that miR-601 expression was significantly associated with TNM stage (*P* = 0.001), lymph node metastasis (*P* = 0.018), lymphatic invasion (*P* = 0.039), and distant metastasis (*P* = 0.028). The expression of miR-601 was not significantly correlated with other clinical parameters (all *P* > 0.05, Table 1), including age, gender, perineural invasion, histological type, tumor depth, and venous invasion.

### Prognostic value of miR-601 expression for patients with GC

**Fig. 2:**
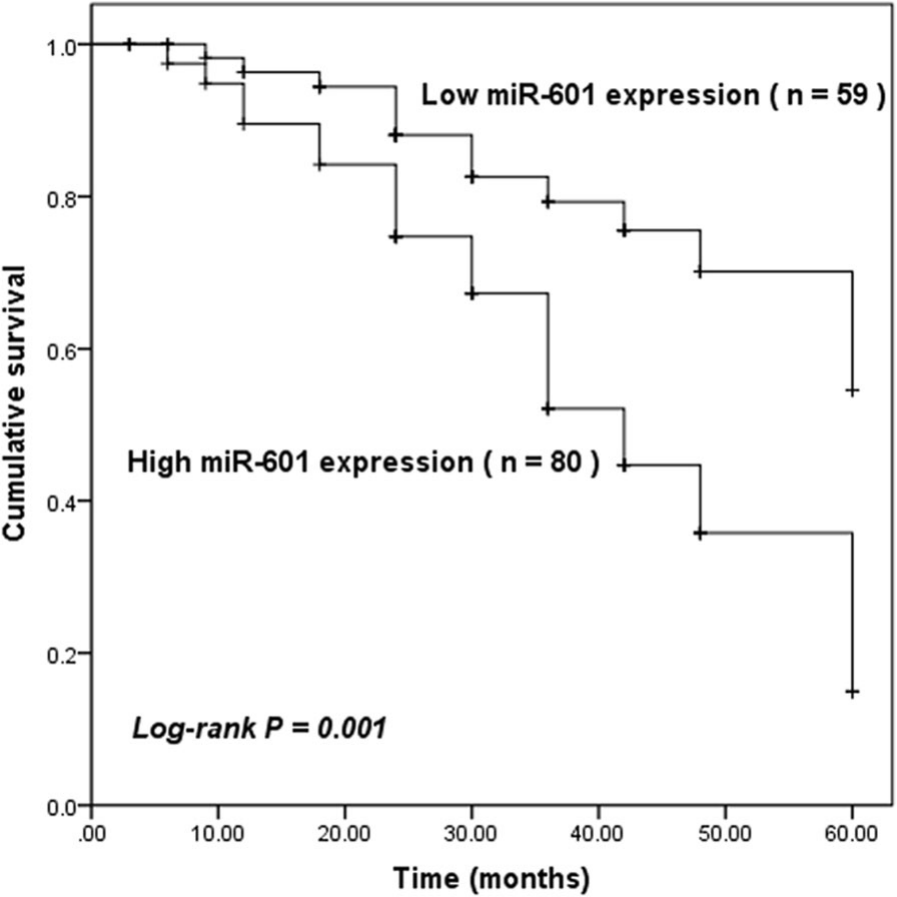
Kaplan-Meier survival analysis for the GC patients based on the expression of miR-601. Patients with high miR-601 expression had shorter survival time than those with low miR-601 expression (log-rank *P* = 0.001)

The prognostic value of miR-601 expression was investigated by using the Kaplan-Meier survival curves. As shown in Fig. 2, we identified that the overall survival rate of GC patients with high miR-601 expression was significantly lower than that of patients with low miR-601 expression (log-rank *P* = 0.001). Furthermore, the clinical parameters and miR-601 expression were included in the multivariate Cox analysis to determine their influence on the overall survival of the cancer patients. The results demonstrated that miR-601 expression (HR = 2.549, 95% CI = 1.236–5.256, *P* = 0.011), TNM stage (HR = 1.962, 95% CI = 1.119–3.440, *P* = 0.019), and distant metastasis (HR = 1.915, 95% CI = 1.047–3.502, *P* = 0.035) were independent prognostic factors for GC (Table 2).

**Table 2:** Multivariate Cox regression analysis for miR-601 in gastric cancer patients

Variables	Multivariate analysis		
	HR	95% CI	*P* value
MiR-601	2.549	1.236–5.256	0.011*
Age	1.175	0.651–2.120	0.592
Gender	0.920	0.522–1.621	0.773
Perineural invasion	0.898	0.514–1.567	0.704
Histological type	1.036	0.599–1.792	0.899
Tumor depth	0.924	0.516–1.653	0.790
TNM stage	1.962	1.119–3.440	0.019*
Lymph node metastasis	1.992	0.692–5.733	0.202
Lymphatic invasion	0.751	0.258–2.183	0.599
Venous invasion	1.738	0.957–3.154	0.069
Distant metastasis	1.915	1.047–3.502	0.035*

*P < 0.05

### Effects of miR-601 on cell proliferation, migration, and invasion in GC cells

**Fig. 3:**
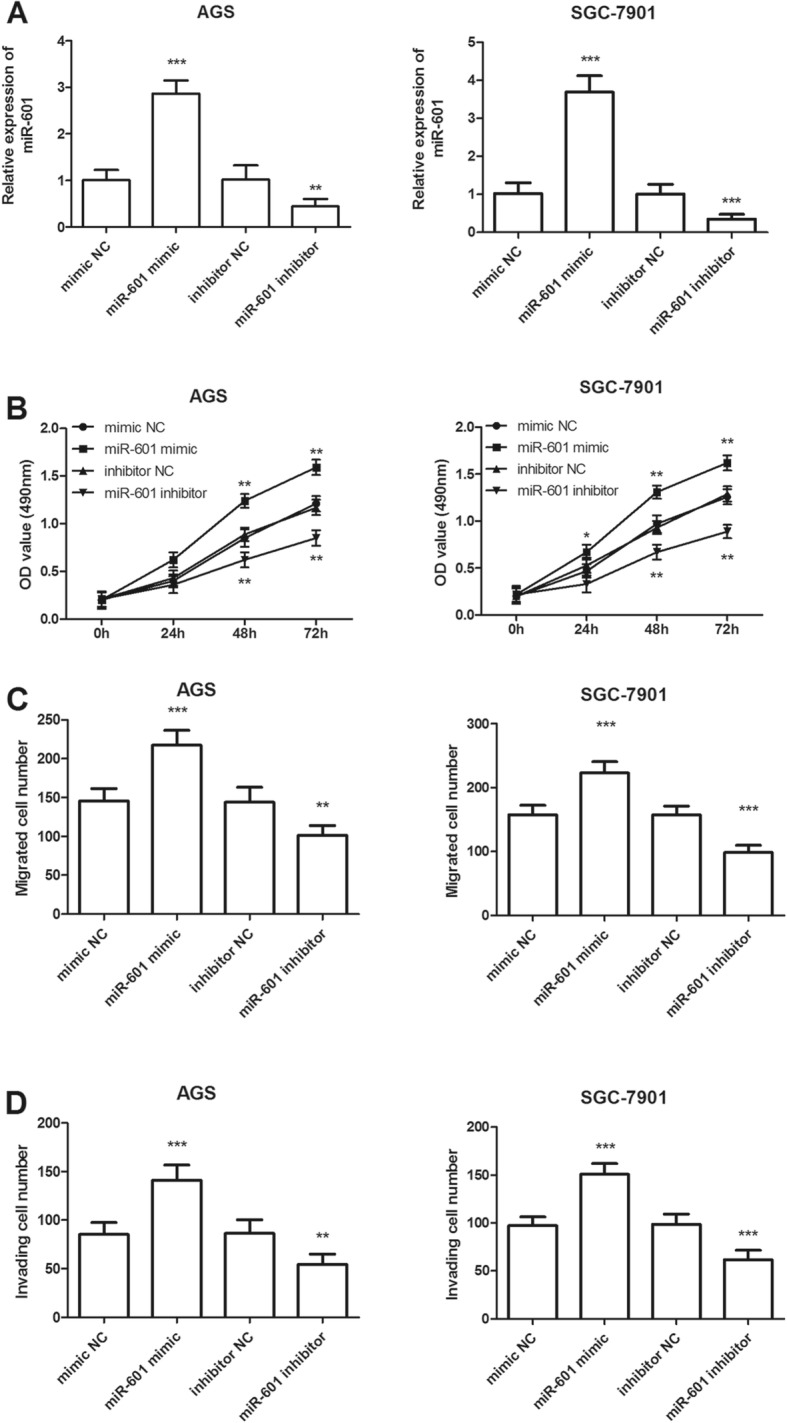
Effects of miR-601 on cell proliferation, migration and invasion in AGS and SGC-7901 cells. **a** In the two cell lines, expression of miR-601 was significantly increased by miR-601 mimic transfection, while transfection with miR-601 inhibitors resulted in lower expression than the corresponding negative control (** *P* < 0.01 and *** *P* < 0.001). **b** Cell proliferation was enhanced by overexpression of miR-601, but was suppressed by knockdown of miR-601 in both AGS and SGC-7901 cells (*P < 0.05 and ** *P* < 0.01). **c** and **d** Overexpression of miR-601 by miR-601 mimic transfection could promote the cell migration and invasion, but the downregulated miR-601 expression could inhibit the cell migration and invasion (**P < 0.01 and ***P < 0.001)

To investigate the role of miR-601 in GC cells, we transfected the AGS and SGC-7901 cells with miR-601 mimic, inhibitor or the corresponding negative controls. qRT-PCR was conducted to evaluate the expression levels of miR-601. As shown in Fig. 3a, miR-601 expression level significantly increased in the cells transfected with miR-601 mimic, but decreased in those with miR-601 inhibitor, compared with the negative controls (all *P* < 0.01). The MTT results suggested that the upregulation of miR-601 significantly promoted cell proliferation as compared with the negative control in both AGS and SGC-7901 cells, while the downregulation of miR-601 significantly suppressed the cell proliferation (all *P* < 0.05, Fig. 3b). As shown in Fig. 3c, the migrated cell number significantly increased in miR-601 mimics transfected cells compared with that in negative controls both in AGS and SGC-7901 cells, while the migrated cell number decreased remarkably in miR-601 inhibitor transfected cells (all *P* < 0.01). Moreover, the invasion assay indicated that overexpression of miR-601 by miR-601 mimic transfection significantly increased the number of invasive cells (all *P* < 0.001), but the silencing of miR-601 by miR-601 inhibitor transfection decreased the ability of invasion of both AGS and SGC-7901 cells (all *P* < 0.01, Fig. 3d).

## Discussion

GC is considered as a major public health problem worldwide, with high morbidity and mortality [15]. Although great progress has been achieved over the last few years in surgical technique, chemotherapy and radiotherapy, the survival rate of GC patients remains unsatisfactory [16, 17]. Better understanding of the mechanisms of GC could be conductive to effective diagnosis and treatment of GC. Numerous studies have identified substantial molecular markers for GC, it is still urgent to find new prognostic biomarkers for establishing targeted strategies to reduce the burden of disease [18, 19]. In recent years, the abnormal expression of miRNAs has been widely detected in diverse tumor samples, and plays an important role in the cancer process [20, 21]. Therefore, it is still of great significance to explore the clinical and functional role of miRNAs, which may provide effective therapy for GC.

MiR-601 is an important member of miRNA family. The abnormal expression of miR-601 has been detected in various human cancers [22]. It is noteworthy that the role of miR-601 as an oncogene or a tumor suppressor varies with the type of cancer. For instance, Cao et al. demonstrated that the miR-601 expression was significantly downregulated in pancreatic cancer (PC) samples, and overexpression of miR-601 inhibited PC cell proliferation and migration [23]. The downregulation of miR-601 was also detected in colorectal cancer (CRC), it was identified to be a potentially promising non-invasive biomarker for the early detection of CRC [24]. Another study also illustrated that miR-601 suppressed the cell growth and invasion in breast cancer via targeting PTP4A1 [25]. On the contrary, the tumor-promoting action of miR-601 has also been detected in some tumor types. Scheffer et al. reported that the expression levels of miR-601 were upregulated in bladder cancer tissues [26]. Furthermore, increased expression of miR-601 was reported in GC compared to normal or adenoma samples [14, 27]. Consistently, the upregulation of miR-601 was confirmed again in a larger cohort of GC patients in the present study. Clinically, a significant association was detected between miR-601 expression and TNM stage, lymph node metastasis, lymphatic invasion and distant metastasis in GC patients, suggesting the important role of miR-601 in the metastasis of GC.

As a result of the alteration of miR-601 expression in GC, its clinical value was further examined in GC prognosis. It was noted that miR-601 expression was an independent prognostic factors for GC, and the overexpression of miR-601 showed significant association with poor overall survival of GC patients. Furthermore, the biological function of miR-601 in GC was further explored in vitro experiment. Most importantly, we showed for the first time that overexpression of miR-601 promoted cell proliferation, migration and invasion in GC cells. Considering the results, we concluded that miR-601 could promote tumor progression in GC. MiR-601 has been reported to be involved in several cell biological behaviors. According to the DNA microarray and global pathway analyses, miR-601 was proved to be associated with cell apoptosis in human lung cancer [28]. Another study reported by Chen et al. suggested that miR-601 was involved in the regulation of hydrogen peroxide (H_2_O_2_) induced apoptosis in pigment epithelium (RPE) cells [29]. In the present study, miR-601 was determined to promote cell proliferation, migration and invasion in GC cells. Considering the results, we concluded that miR-601 could promote tumor progression in GC.

The role of miR-601 in tumor progression has been reported in several types of cancers. Hu et al. showed that miR-601 was down-regulated in breast cancer tissues and ectopic overexpression of miR-601 inhibited tumor cell proliferation, migration, and invasion [25]. Another study in esophageal squamous cell carcinoma (ESCC) demonstrated that HDAC6 was the target gene of miR-601, and miR-601 suppressed tumor proliferation and metastasis by down-regulating HDAC6 expression [30]. Interestingly, HDAC6 was reported to be downregulated in GC tissues and was an independent predictor of the outcome of GC patients [31]. In our present study, it was observed that miR-601 was up-regulated in GC patients and involved in the progression of GC. Combined with the previous evidence and the present results, we speculated that miR-601 serves as an oncogene in GC, and might promote tumor cell proliferation, migration, and invasion via regulating the expression of HDAC6.

In conclusion, our study showed that the expression level of miR-601 was dramatically up-regulated in GC tissues and cell lines. Overexpression of miR-601 was associated with poor prognosis of GC and promoted proliferation, migration, and invasion of GC cells. The present study provides functional evidence for the first time that miR-601 might be a potential therapeutic target in GC treatment. However, the precise molecular mechanisms behind the altered expression of miR-601 in GC progression is still not very clear, and further research is needed.

## Data Availability

All data generated or analysed during this study are included in this published article.

## Abbreviations

CRC: Colorectal cancer
DMSO: Dimethyl sulfoxide
ESCC: Esophageal squamous cell carcinoma
FBS: Fetal bovine serum
GC: Gastric cancer
H_2_O_2_: Hydrogen peroxide
MiRNAs: MicroRNAs
mRNAs: messenger RNAs
PC: Pancreatic cancer
qRT-PCR: Quantitative real-time polymerase chain reaction
RPE: pigment epithelium
SD: Standard deviation
